# Venomics Reveals a Non-Compartmentalised Venom Gland in the Early Diverged Vermivorous *Conus distans*

**DOI:** 10.3390/toxins14030226

**Published:** 2022-03-19

**Authors:** Jutty Rajan Prashanth, Sebastien Dutertre, Subash Kumar Rai, Richard J. Lewis

**Affiliations:** 1 Institute for Molecular Bioscience, University of Queensland, St. Lucia, QLD 4072, Australia; prashanth.rajan185@gmail.com (J.R.P.); subash.rai@uq.edu.au (S.K.R.); 2 IBMM, Université Montpellier, CNRS, ENSCM, 34095 Montpellier, France; sebastien.dutertre@umontpellier.fr

**Keywords:** proteomics, transcriptomics, evolution, defensive venom, conotoxins

## Abstract

The defensive use of cone snail venom is hypothesised to have first arisen in ancestral worm-hunting snails and later repurposed in a compartmentalised venom duct to facilitate the dietary shift to molluscivory and piscivory. Consistent with its placement in a basal lineage, we demonstrate that the *C. distans* venom gland lacked distinct compartmentalisation. Transcriptomics revealed *C. distans* expressed a wide range of structural classes, with inhibitory cysteine knot (ICK)-containing peptides dominating. To better understand the evolution of the venom gland compartmentalisation, we compared *C. distans* to *C. planorbis*, the earliest diverging species from which a defence-evoked venom has been obtained, and fish-hunting *C. geographus* from the *Gastridium* subgenus that injects distinct defensive and predatory venoms. These comparisons support the hypothesis that venom gland compartmentalisation arose in worm-hunting species and enabled repurposing of venom peptides to facilitate the dietary shift from vermivory to molluscivory and piscivory in more recently diverged cone snail lineages.

## 1. Introduction

Venoms have evolved multiple times across different lineages to aid predation, defence and competitor deterrence [1]. They are injected into the target species through specialised apparatuses that have evolved under ecological pressures together with the venom peptides they deliver [2,3]. The biological activity of venom peptides and the mechanisms to express and inject venom are influenced by ecological interactions with prey and predators [4,5,6], with some species of molluscs (cone snails) and insects (assassin bugs) able to deploy separately evolved predatory and defensive venoms [7,8].

Cone snails are circumglobal venomous marine gastropods found in tropical waters that use venoms for predation and defence. More than 800 species of cone snails have been identified mostly in the Indo-Pacific region, including ~700 species of the genus *Conus* [9]. Ancestral and most extant cone snail species are worm-hunters, with mollusc- and fish-hunting species having evolved more recently [10]. Some cone snail species, including the fish-hunting *Conus geographus* and the mollusc hunting *Conus marmoreus,* inject different venoms for defence and predation [7]. These distinct defensive and predatory venoms are injected using the same radula tooth envenomation apparatus but are differentially expressed from different sections of the venom duct [11]. Based on the venom duct analysis of *C. geographus*, defence-evoked venoms are typically expressed in the proximal section (near the venom bulb) and those used for predation in the distal section (near the proboscis) [7]. However, in the worm-hunting *Conus planorbis*, the defensive venom peptides were not restricted to the proximal section, despite variable venom production along the duct [12]. In contrast, more recently diverged worm-hunting species, such as *Conus vexillum* [13], produced defensive venoms that dominated only the proximal duct. We hypothesised that the defensive use of venom first arose in ancestral worm-hunting snails and was later repurposed in a compartmentalised venom duct to facilitate the dietary shift to molluscivory and piscivory [7].

To better understand when venom gland compartmentalisation might have evolved in cone snails, we studied the venom of the earliest diverging extant Conidae, *Conus distans*, from the *Fraterconus* subgenus [9,14]. The venom of this species is little studied, with previous assay-guided efforts only identifying proteins between 24 kDa and 25.5 kDa that blocked calcium influx associated with neurotransmitter release in the hippocampus of rats and bovine chromaffin cells [15,16] and a novel excitatory peptide, DiXIXA, possessing a rare triple cysteine motif [17].

Consistent with its placement within a basal lineage, we demonstrate here that the *C. distans* venom gland lacked distinct compartmentalisation. Transcriptomics revealed *C. distans* expressed a wide range of structural classes, with inhibitory cysteine knot (ICK)-containing peptides dominating. We compared *C. distans* to *C. planorbis*, the earliest diverging species from which a defence-evoked venom has been obtained [12], and *C. geographus*, a fish-hunting cone snail from the *Gastridium* subgenus that injects highly distinct defensive and predatory venoms [7]. These data support the hypothesis that venom gland compartmentalisation likely arose in an ancestral worm-hunting species and facilitated the dietary shift from vermivory to molluscivory and piscivory in cone snails.

## 2. Results

### 2.1. The Venom Gland of C. distans Is Not Compartmentalised

**Figure 1:**
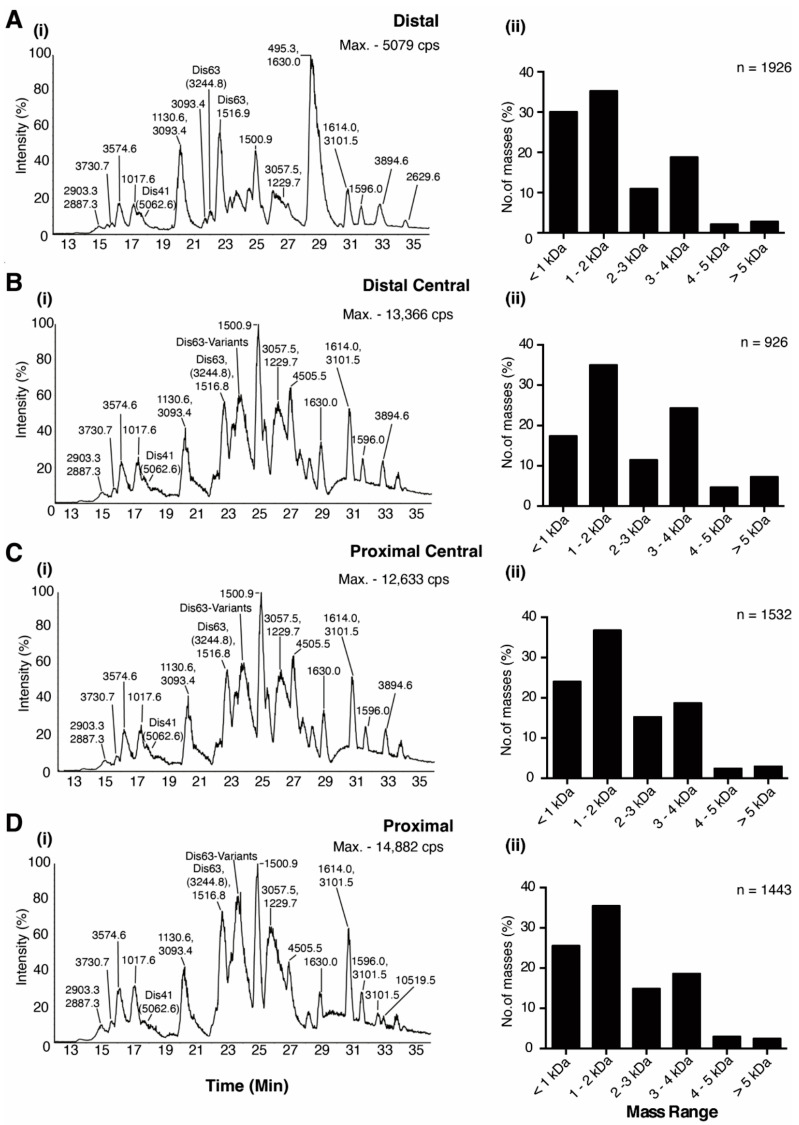
Proteomics of venom gland sections of *C. distans*. (**A**(**i**)**–D**(**i**)) show LC-ESI-MS profiles of the four venom duct sections, with major masses labelled. (**A**(**ii**)**–D**(**ii**)) show mass distributions for the corresponding sections.

To examine whether venom expression in *C. distans* varied along the venom gland, we performed LC-ESI-MS experiments on venom extracted from the distal (closest to the proboscis), distal central, proximal central and proximal (closest to the venom bulb) venom duct of *C. distans* (Specimen-1). Comparing base peak chromatograms (BPCs), we observed that the elution time of all major masses were similar across the four sections of the gland (Figure 1). Manual reconstruction of the masses within each co-eluting peak confirmed minimal qualitative or quantitative differences between venom peptides secreted from each section, except for several late eluting minor peaks (Figure 1A(i)–D(i)). We also reconstructed the masses using the LC-MS reconstruction and observed that the mass distributions within the four sections were similar, with the venom profile of *C. distans* dominated by 1–4 kDa peptides (Figure 1A(ii)–D(ii)). Each section also had a significant number of masses <1 kDa, which were not characterised further but may include small molecules similar to those discovered in another early diverging species, *Conus imperalis* [18]. We could not detect any masses corresponding to DiXIXA, the only peptide isolated from this species previously [17], but were able to identify two peptide masses (Dis41 and Dis63; Figure 1) that corresponded to two novel conotoxins we identified in the venom gland transcriptome of another *C. distans* specimen.

**Figure 2:**
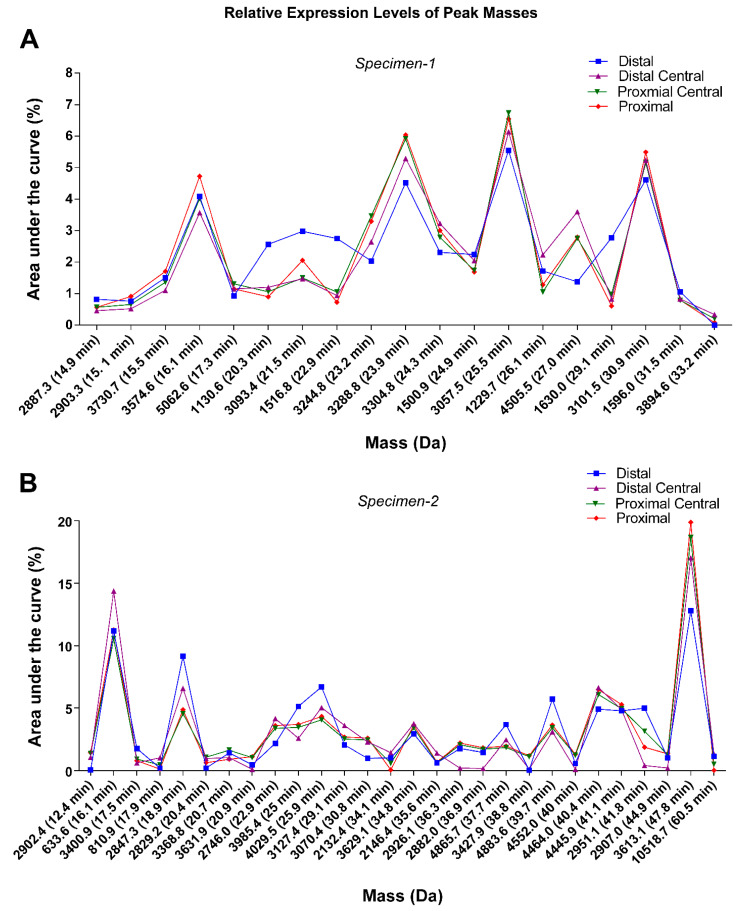
Relative expression levels of dominant masses in *C. distans* venom gland sections. Comparison of relative expression levels (% total area under the curve) of masses across the four sections of the venom gland. (**A**) LC-ESI-MS chromatograms of (**A**) Specimen 1 and (**B**) Specimen 2 were run for 65 min and 100 min, respectively.

To confirm whether *C. distans* has a non-compartmentalised venom duct, we analysed a second specimen obtained from a different geographical location (Specimen 2). Again, the BPC revealed that the expression of major masses along the venom gland was uniform (Supplementary Figure S1). We manually reconstructed the masses in each major peak for both specimens and compared the expression levels across the four sections of their respective glands. Despite substantial differences in the specific masses detected between the two specimens, the expression levels of major common masses were similar across the length of the gland in both species, demonstrating that the venom gland of *C. distans* is indeed non-compartmentalised (Figure 2).

### 2.2. C. distans Venom Gland Transcriptome

**Figure 3:**
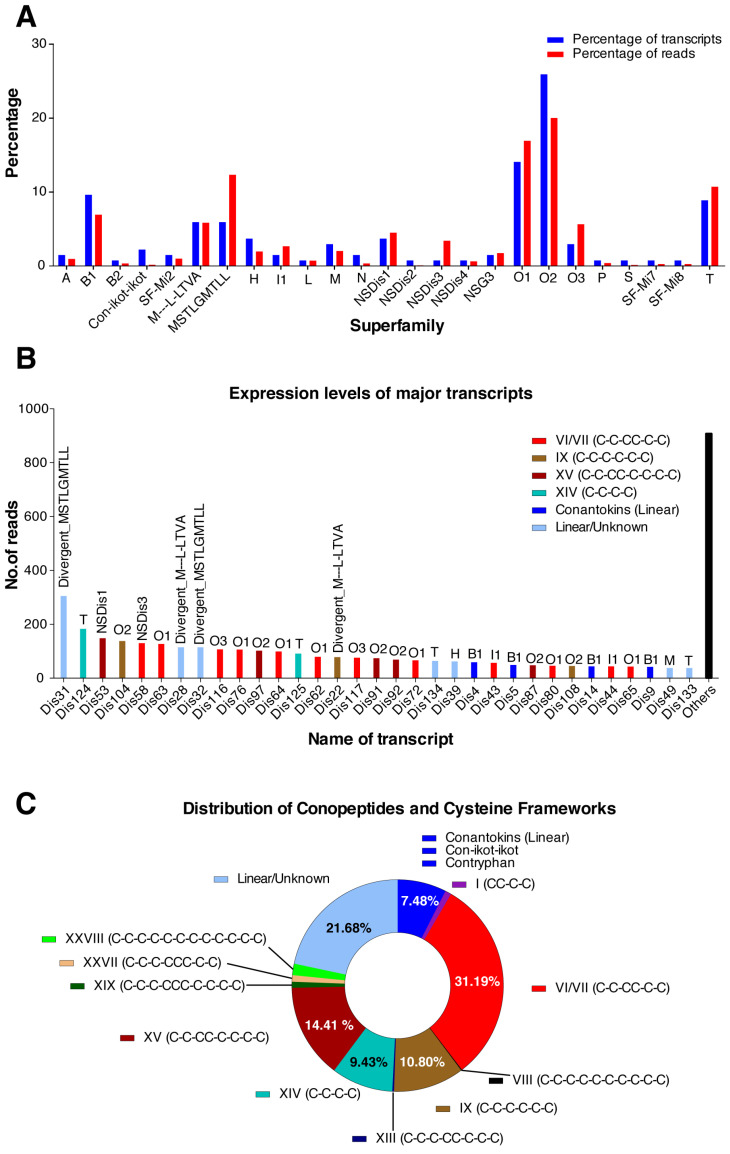
Venom gland transcriptome of *C. distans*. (**A**) Expression levels of various superfamilies in the terms of number of transcripts and sequence reads. (**B**) Highly expressed transcripts (expression level >1% of total reads) in *C. distans*. The superfamily and predicted cysteine framework are indicated. (**C**) The distribution of cysteine frameworks in the transcriptome dataset.

We sequenced the venom gland transcriptome of the third *C. distans* (Specimen 3) using 454-pyrosequencing to identify the expressed venom peptide sequences. Specimen 3 produced 209,346 total reads, including 183,763 reads with a sequence quality score cut-off of >30 and an average length of ~332 nucleotides. Trimmed reads (see Materials and Methods) contained 135 conotoxin transcripts belonging to 25 different superfamilies, including four putative novel superfamilies. Expression levels of various superfamilies varied considerably, with the O_2_ and O_1_ superfamilies the most highly expressed (~20% and ~17%, respectively). Divergent superfamilies reported in *Conus californicus* [19] and novel superfamilies NSDis1 and NSDis3 discovered here were also found at high levels, along with the B1 (conantokins), O_3,_ and T superfamilies (Figure 3A). A total of 33 highly expressed transcripts, defined as those contributing at least 1% of total conotoxin reads (≥38 reads, total read count = 3796), accounted for ~76% of all conotoxin reads (Figure 3B). A majority of these highly expressed transcripts belonged to the dominant superfamilies O_2_, O_1_, B1 and T (Figure 3A,B). Alignments of the different sequences are presented in Supplementary Figures S1–S5.

ICK-containing framework VI/VII (C-C-CC-C-C; 6/7 cysteines) (~31.2%), framework IX [20] (C-C-C-C-C-C; 6 cysteines) (~10.8%) and framework XV peptides (C-C-CC-C-C-C-C; 8 cysteines) (~14.4%) were the most common in *C. distans*. Additionally, linear conantokin-like peptides (7.3%), and framework XIV (C-C-C-C; 4 cysteines) (9.4%) were also highly expressed (Figure 3B,C). Rare triple cysteine motifs associated with framework XIX (C-C-C-CCC-C-C-C-C; 10 cysteines) and framework XXVII (C-C-C-CCC-C-C; 8 cysteines) were also discovered, albeit at lower levels. The framework XIX peptide DiXIXA, which is the only peptide to have been isolated and sequenced from this species previously [17], was moderately expressed in our transcriptome (35 reads). Framework I peptides (CC-C-C; 4 cysteines) from the A-superfamily closely associated with α-conotoxins and framework VIII peptides (C-C-C-C-C-C-C-C; 8 cysteines) from the S-superfamily were also found at lower levels (Figure 3C).

The range of superfamilies and frameworks in the transcriptome suggests that the venom of *C. distans* is pharmacologically diverse. BLAST results revealed that *C. distans* sequences were broadly divergent from other conotoxins, though some exceptions were found (see Table 1). For example, the M-superfamily sequence Dis46 was 100% similar to Im6.7 from *C. imperialis*, another basal species. Additionally, several highly divergent sequences were identified with homology to *C. californicus*, Turrid and Terebrid toxins, indicating they might be used across the wider Conoidea family. *C. distans* sequences also showed homology to sequences from fish-, mollusc- and worm-hunting species, including sequences with homology to SF-Mi2 peptides from *C. miles* [21], NSG3 from *C. geographus* [7], and framework IX peptides from the P-superfamily from *Conus gloriamaris* and *Conus textile* [22]. Among the major pharmacological classes, two putative α-conotoxins (nAChR blockers) with a CC-C-C framework and extended N-termini were uncovered with the rare CC-X_4-_C-X_8_-C pattern. An S-superfamily framework VIII peptide (Dis121) with similarity to σ-GVIIIA (5-HT_3_ receptor antagonist) [23] and α-RVIIIA [24] was also discovered in *C. distans*, along with several highly expressed ω-conotoxin-like (Ca^2+^ channel antagonists) sequences, whereas δ-, μ- or μO-conotoxin-like sequences likely to target the Na^+^ channel were not detected. Finally, several linear conantokin-like peptides (Dis3-Dis16) were found in high levels (>1% of total reads) (Figure 3) and sequences belonging to the con-ikot-ikot superfamily (Dis17-Dis19) were expressed at low levels in *C. distans* (Figure 3A). An overview of the diversity of conotoxins in *C. distans* is provided in Table 1.

### 2.3. Identification of Transcriptomic Sequences in Venom Duct Extracts

To determine which transcriptomic sequences are detectable in the venom, we performed MS experiments and matched transcriptomic and proteomic data. Briefly, native venom samples along with reduced, alkylated and enzymatically digested samples were analysed by LC-ESI-MS and MS/MS. MS/MS data were matched to the transcriptome sequences using ProteinPilot and only sequences identified with >99% confidence were considered. Masses of the predicted mature peptides were then compared to the LC-ESI-MS experiments to identify possible PTMs. A total of 48 sequences from 16 superfamilies were validated by MS/MS. Surprisingly, 35 highly expressed transcripts and their variants were not found as major components in the venom. The identified sequences along with predicted PTMs are shown in Table 2.

### 2.4. Comparison of C. distans with C. planorbis

*C. planorbis* has been placed into the *Strategoconus* subgenus of *Conus* based on the molecular phylogenetic classification [9]. *C. planorbis* remains the earliest diverging species from which a defensive venom has been collected [12]. However, Jin et al. reported that unlike the more recently evolved species [7,13], the expression of defensive venom peptides in *C. planorbis* was not restricted to the proximal sections [12]. Therefore, we hypothesised that the venom gland of *C. planorbis* might represent a transitionary state between non-compartmentalised venom glands in *C. distans* and the compartmentalised glands found in piscivorous species such as *C. geographus*. To understand this comparison better, we performed additional MS experiments on three specimens of *C. planorbis* and one specimen of *C. geographus*, obtained from the northern Great Barrier Reef.

**Figure 4:**
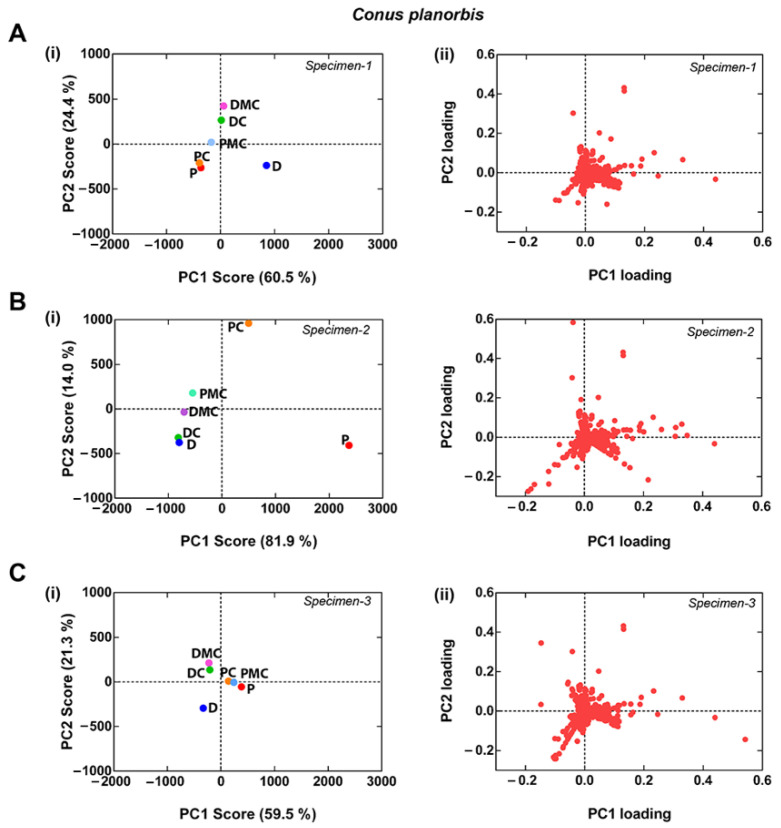
Principal Component Analysis (PCA) analysis of *Conus planorbis.* (**A**(**i**)–**C**(**ii**)) scores plots indicate the diversity of venom gland segments within the *C. planorbis*, and (**A**(**ii**)–**C**(**ii**)) loading plots show families of correlated variables. Specimen 1 includes the highest number of mass peaks (430) among three specimens in PCA. P: Proximal, PC: Proximal Central, PMC: Proximal Middle Central, DMC: Distal Middle Central, DC: Distal Central, and D: Distal.

To improve spatial resolution, the venom gland was divided into six sections and MS analysis performed as described for *C. distans*. Principal Component Analyses (PCA) [25] were then used to compare the variance between each section of the venom gland. The venom gland of *C. planorbis* shows differentiation along the gland, as previously reported [12], though the levels of variance differ between individuals (Figure 4). Specimen 3 was least differentiated, Specimen 2 was most differentiated, while Specimen 1 showed intermediate levels of differentiation. Specimen 1 had the highest number of masses and was used as the reference specimen for *C. planorbis*.

**Figure 5:**
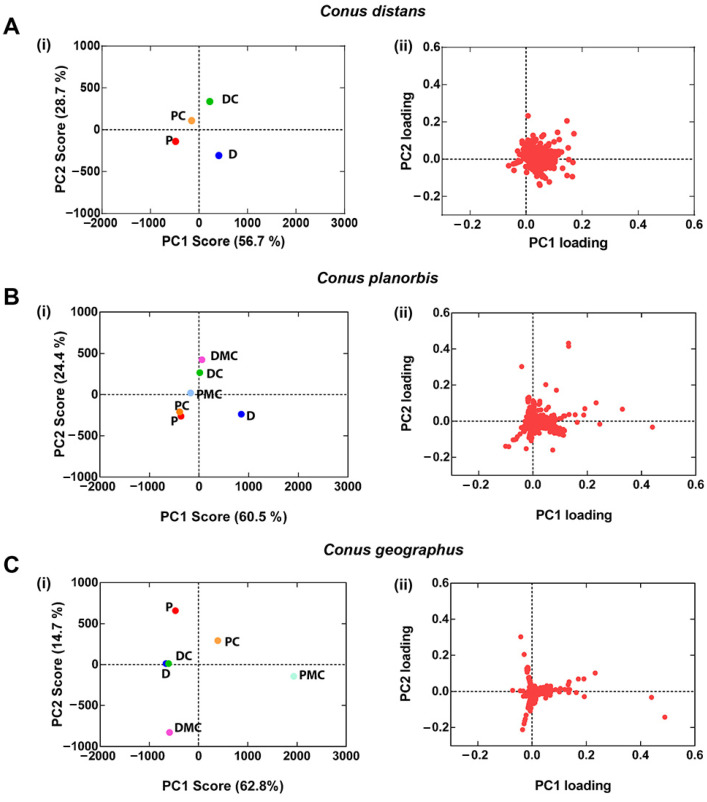
PCA analysis of venom gland segments of *C. distans, C. planorbis* and *C. geographus*. (**A**(**i**)–**C**(**i**)) PC1 and PC2 scores plots show the separation of venom gland segments in indicated species and (**A**(**ii**)–**C**(**ii**)) PC1 and PC2 loadings plots show the families of correlated variables. Percentages of variance used in PCA of each species are indicated in the scores plot. P: proximal, PC: proximal central, PMC: proximal middle central, DMC: distal middle central, DC: distal central, and D: distal.

Figure 5 compares the PCA results of *C. distans* Specimen 2 with the *C. geographus* specimen and *C. planorbis* Specimen 2. The ion clustering in the different species (Figure 5 B) suggests that the *C. planorbis* venom gland represents an intermediate (transitionary) phase between the non-compartmentalised venom gland found in *C. distans* and the highly compartmentalised venom gland of *C. geographus*.

## 3. Discussion

It has been established that mollusc- and fish-hunting cone snails can inject distinct and separate defensive and predatory venoms [7]. Defensive use of venom has been demonstrated in mollusc-, fish- and worm-hunting species [7,13]. From these observations, it was hypothesised that ancestral defensive venom peptides used by worm-hunting species to deter molluscs and fish predators were later repurposed and compartmentalised to facilitate the transition to mollusc- and fish-hunting [7]. However, it remains unclear when compartmentalisation of the venom gland occurred within the genus *Conus* to enable the secretion and injection of distinct venoms for different ecological roles.

Since *C. distans* belongs to the stem group of the genus *Conus*, we studied the expression of venom peptides across the gland in the species and compared it to another early diverging species, *C. planorbis,* as well as a more recently derived fish-hunter, *C. geographus*. Our proteomic data showed that overall expression of venom remained similar throughout the gland with only minor quantitative variations detected (Figure 2). This contrasts with later diverged species previously studied, supporting the hypothesis that ancestral cone snails had a non-compartmentalised venom gland. Thus, these data suggest *C. distans* diverged prior to the appearance of predatory and defensive venom compartmentalisation and likely uses the same venom for both predation and defence. Given that specialised mollusc- and fish-hunting are derived traits that have originated within the genus *Conus* [14], venom gland compartmentalisation and stimulus-dependent venom deployment could have arisen uniquely within this genus. The *C. distans* venom provides an opportunity to study quasi-ancestral conotoxin structure and pharmacology before predatory and defensive venoms diverged.

We surveyed the venom composition of *C. distans* by sequencing its venom gland transcriptome on the 454-pyrosequencing platform, with RNA-seq data as described earlier [13,26]. The venom gland transcriptome was characteristically diverse in terms of superfamilies, cysteine frameworks and probable pharmacology. A total of 135 transcripts belonging to 25 superfamilies were recovered, with early evolved superfamilies such as O_1_, O_2_ and T dominating (Figure 3). In addition, several divergent superfamilies originally reported in *C. californicus* were also expressed at high levels as well as four novel superfamilies. Structurally, the transcriptome was dominated by peptides containing frameworks VI/VII, IX or XV, which are characterised by their ICK motif (Figure 3C). Interestingly, a framework VI/VII peptide from the H-superfamily was highly similar to a teretoxin, while some framework IX peptides were similar to *C. californicus* and turrid toxins (Table 1). These toxins likely represent toxins inherited from an ancestral toxoglossate mollusc, given their prevalence across separate genera. Several conotoxins and conopeptides that were similar to others found in mollusc- and fish-hunting cone snails were also identified. Conantokins (NMDA receptor antagonists) [27], con-ikot-ikots (AMPA receptor antagonists) [28] and a contryphan [29] are all expressed by *C. distans*. Interestingly, these toxin families were found to be prominent in the predatory venom of the fish-hunting *C. geographus,* suggesting potential repurposing for predation [7]. Two different peptide classes containing unique triple cysteine motifs were also discovered. One of them, DiXIXA, is the only peptide to have been isolated from this species so far, and elicits excitatory activity when injected in mice [17]. The other peptides belonged to SF-Mi2, a recently discovered superfamily reported originally in *C. miles* [21]. Little is known about the structure and the activity of this group of toxins. Thus, the venom gland transcriptome reveals that the venom of *C. distans* contains a number of novel conotoxins whose pharmacology is yet to be characterised.

**Figure 6:**
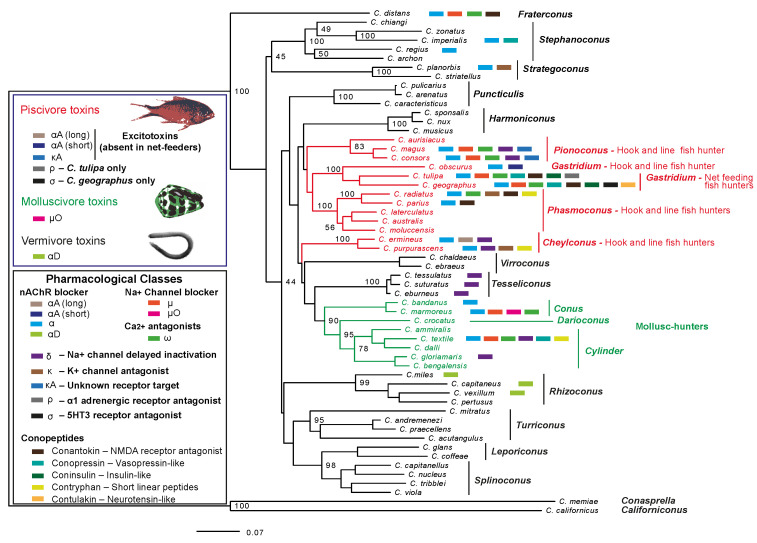
Overview of the major venom components of Conidae. The phylogenetic reconstruction was adapted from Puillandre et al. [14]. Lineages in green indicate mollusc-hunting species and lineages in red indicate fish-hunting species. All other lineages are predominantly comprised of worm-hunters except for divergent species such as *Conus californicus*, which can prey on fish, worms and molluscs.

We also identified two putative α-conotoxins with a novel 4/8 cysteine arrangement. Though the pharmacological and evolutionary significance of this subtype of α-conotoxins has not been defined, α-conotoxins are widely used in defence across the Conidae [7,12,13]. We also found putative ω-conotoxins among the transcriptomic sequences exhibiting a sequence similarity to ω-conotoxins from fish-hunting species. However, using BLAST we could not find any obvious δ-, μ- or μO-like sequences [30,31,32]. A third type of μ-conotoxins, framework V, containing peptides belonging to the T-superfamily, has also been identified [33] but could not be detected either. κ-Conotoxin K^+^ channel blockers are presumed to have convergently evolved from a range of different structural folds [34]. While *C. purpurascens* employs PnVIIA, a framework VI/VII peptide from the O1-superfamily [35], a kunitz-containing protein was proposed to act similarly in *C. striatus* [36]. While some sequences in our transcriptome with the framework IX were similar to *C. californicus* sequences that contained a kunitz-motif [37], a search across the Conserved Domain Database retrieved no hits for the kunitz-domain and no other sequences with similarities to κ-conotoxins were identified. While the presence of highly divergent δ-, μ- or κ-conotoxins in *C. distans* cannot be excluded, it appears that these would be no more than minor components of the venom. Given there are intraspecific variations within cone snail species, sequencing the venom gland transcriptomes of more specimens of *C. distans* would help to further elucidate intraspecific differences in venom expression. Nonetheless, our data suggests early diverging cone snails expressed sequences ancestral to modern α- and ω-conotoxins found in mollusc- and fish-hunting cone snails. Figure 6 provides an overview of how *C. distans*’ venom gland transcriptome compares with other cone snail species when placed within its phylogenetic context, as determined by Puillandre et al. [14].

Previous screening attempts using *C. distans* venom have identified few conotoxins with potent biological activity. Worm, mollusc and/or fish assays specifically designed to identify molecules targeting ecologically relevant species are expected to identify more bioactive peptides. Interestingly, *C. distans* venom is unique in having a large proportion of low molecular weight molecules <1 kDa. The total ion chromatograms (TICs) from LC-ESI-MS experiments from both *C. distans* specimens did not show any major peaks despite numerous attempts to optimise the sample runs with different LC-MS conditions. Unfortunately, we were unable to elicit a defensive response from *C. distans* when stimulated by a predator such as *C. marmoreus* or *C. textile*, as previously demonstrated in another early diverging species, *C. imperialis*. Given the relatively large size and shell strength of *C. distans*, the shell alone might offer sufficient protection from most predators for this species and *C. distans* may potentially lack a defensive venom. We were also unable to find suitable prey for *C. distans* to collect the predation-evoked venom to establish if the full complement of venom peptides, or only a specific subset, are expressed during predation. The low expression levels of masses that correspond to the typical conotoxin mass range raises the possibility that *C. distans* may have evolved defensive and predatory strategies in the absence of obvious venom gland compartmentalisation.

To further understand the evolution of the venom gland within the genus *Conus*, we compared *C. distans* with *C. planorbis* and *C. geographus*. *C. planorbis* is the earliest diverging species from which a defence-evoked venom was obtained, notably in the presence of *C. marmoreus*, a molluscivore cone snail [12]. Uniquely among species from which defensive stings have been collected, expression of defensive venom peptides in *C. planorbis* was not restricted to the proximal section [12]. We used *C. geographus* as a reference species, as the use of defensive and predatory venoms in this species has been well established [7,38]. Our comparison here using PCA analysis showed that the expression of peptides across the venom gland of *C. planorbis* is more compartmentalised than *C. distans* but less compartmentalised than *C. geographus*. This suggests that the venom gland of *C. planorbis* represents an evolutionary stage that is intermediate between the non-compartmentalised *C. distans* venom gland and the highly compartmentalised glands observed in more recently evolved worm and fish-hunting species [13].

## 4. Materials and Methods

### 4.1. Sample Collection, RNA Extraction and Sequencing

Specimens of *C. distans* were collected from Gould Reef in the central Great Barrier Reef (Queensland, Australia) and maintained alive in marine aquaria before use. One specimen was carefully dissected on ice and the venom gland placed in an Eppendorf tube containing 1 mL of TRIZOL (Invitrogen, Carlsbad, CA, USA) and total RNA extracted following the manufacturer’s instructions. Next, mRNA was purified using the Oligotex mRNA kit and ~200 ng was sequenced by the Australian Genome Research Facility (AGRF, Brisbane) on a Roche 454-GS FLX plus titanium sequencer. Sequencing yielded 209,346 reads with an average read length of ~350 bp after filtering poor quality reads.

### 4.2. Transcriptomic Analysis

Transcriptome analysis was performed as previously described [26]. Briefly, raw reads were filtered based on quality scores (Quality >30; 1 in 1000 incorrect base call frequency) and sequences possessing an archetypal conotoxin precursor structure [39] were sorted into superfamilies using Conosorter. Unclassified sequences with >50 A.A., a read count ≥2, a class score >1, and superfamily score >0 were also included. Reads with incomplete signal sequences, unrecognised amino acids, frameshifts or truncations, and duplicated sequences were discarded. After discarding housekeeping proteins, the remaining sequences were clustered by their signal region and classified using BLASTp. ConoPrec [40] was then used to place conotoxin-like sequences into known superfamilies or to designate novel Superfamilies based on a signal sequence similarity cut-off of 53.3% [26]. Sequences were then manually inspected, aligned and visualised using Ugene [41].

### 4.3. Sample Collection for Mass Spectrometry

*C. distans* specimen 1 was collected from Lady Musgrave Island on the southern Great Barrier Reef and *C. distans* specimen 2 was collected off Cairns in the northern Great Barrier Reef. Both were maintained in an aquarium with a 12 h day/night cycle until use. All three *C. planorbis* specimens and *C. geographus* were collected off Cairns in the northern Great Barrier Reef.

### 4.4. Venom Extraction

Specimens were dissected on ice and the venom gland removed intact and unravelled carefully to avoid breakage. The gland was then divided into four equal lengths of distal (near the proboscis), distal central, proximal central and proximal (near the venom bulb) venom duct. Venom from each section was stripped into 500 μL of 30% acetonitrile (ACN)/0.1% formic acid (FA), vortexed for 1 min, sonicated for 30 s and centrifuged on a bench-top machine at 12,000× *g* for 10 min. The supernatant was collected, and the pellet was subjected to two additional rounds of extraction, as described above. Supernatants were pooled, lyophilised overnight, and stored at −20 °C prior to use.

### 4.5. Reduction, Alkylation and Enzymatic Digest of Venom Samples

Powdered venoms were resuspended in 1 M (NH_4_)_2_HCO_3_ at pH 11 (1 μg/μL) and reduction and alkylation solution (97.5% ACN, 2% iodoethanol, 0.5% triethylphospine) added in a 1:1 ratio, followed by incubation at 37 °C for 2 h [42]. MS-grade trypsin and endoproteinase-GluC (Promega, Madison, WI, USA) dissolved in 100 μL of 40 mM NH_4_HCO_3_ were then added to speed-vac-dried sample at a protease-to-protein ratio of 1:100 and incubated overnight at 37 °C. Digested samples were dried on a speed-vac prior to use.

### 4.6. Venom Gland Mass Spectrometry (LC-ESI-MS and MS/MS)

LC-ESI-MS and MS/MS were performed on native and reduced-alkylated-digested samples using a 5600 tripleTOF mass spectrometer with a quadruple TOF system equipped with a TurboV DuoSpray (ABSCIEX, Ontario, Canada) source set to 5300 V and 500 °C. Native and processed venoms were dried to remove buffer, resuspended in 1% formic acid (FA), vortexed and centrifuged at 12,000× *g* for 10 min to remove particulates. Samples were separated on a ZORBAX 300SB-C18 (2.1mm × 100 mm × 1.8 µm) column using a Shimadzu 30 series HPLC system (Shimadzu, Kyoto, Japan). For LC-ESI-MS experiments on *C. distans* specimen 2, *C. planorbis* and *C. geographus* samples, samples were eluted at 0.2 mL/min with a gradient of 1% Solvent A (0.1% FA) to 40% Solvent B (40% acetonitrile/0.1% FA) over 80 min. Full-scan MS data was accumulated over 500 ms with cycle time set to 0.525 s and a mass range of 350–2400 Da. For LC-ESI-MS/MS for *C. distans* specimen 1, samples were eluted at 180 μL/min with a linear gradient of 1–40% over 45 min. The columns were then flushed over 15 min with a linear gradient increasing to 98% buffer B. Separated samples were injected using the TurboV ion spray source with the ionspray voltage of the source set to 5300 V and the temperature to 450 °C. Full-scanning MS data was accumulated over 100 ms followed by full-scan product ion data in the high-resolution information dependent acquisition (IDA) mode. The total duration of the full- scan MS was 60 min, consisting of 1645 cycles of 2.15 s per cycle of full-scan MS. A rolling collision energy based on precursor ion *m/z* was used to generate product ions for the TOF MS/MS. The mass range was set to 300–1800 (*m/z*) for TOF MS mode and 80 –1400 (*m/z*) for full-scan TOF MS/MS mode. Product ions with charge states between 2 and 5, with a minimum intensity of 150 cps, were recorded, and isotopes within 4 Da were binned, though former target ions were retained. To capture the mass range of undigested venom samples more thoroughly, with a particular focus on higher molecular weight components in the venom, we performed LC-ESI-MS experiments on native *C. distans* specimen 1. The LC gradient was set to 2–40% B over 30 min with a flow rate of 0.5 μL/min. Full- scan MS was performed over 54 min, comprising of 3161 cycles of 1.002 s per cycle. Full- scan TOF MS data was obtained over a mass range of 400–2000 Da and accumulated over 1 s.

### 4.7. MS and MS/MS Data Analysis

MS data analysis was performed using Analyst 1.6 and Peakview 2.1 (ABSCIEX, Ontario, Canada). Masses were reconstructed using the LC-MS reconstruct option with a mass range of 400–12,000 Da, a tolerance of 0.2 Da and the S/N ratio threshold of 10. Masses of major peaks were confirmed manually. MS/MS data analysis was performed using ProteinPilot 4.1. Briefly, theoretical spectra generated from transcriptomic sequences were matched to MS/MS spectra from proteomics using the paragon algorithm. Only sequences assigned to spectra at a confidence value >99% were analysed.

### 4.8. Principal Component Analysis (PCA) of LC-ESI-MS Data

Unprocessed LC-ESI-MS data were imported into MarkerView™ Software (v1.3.1) to generate a list of unique masses for each venom gland section. Peaks were detected using a peak width of <100 scans, a noise threshold of 10 and retention time between 10–65 min, with a maximum of 8000 peaks generated for each dataset. Peaks from different samples were aligned and filtered using a retention time tolerance of 0.5 min and mass tolerance of 25 ppm. Unsupervised PCA using Pareto scaling was performed on monoisotopic masses >400 Da. The generated PC1 and PC2 score and loading values were plotted on GraphPad Prism (v7).

## 5. Conclusions

In conclusion, our study of the basal lineage cone snail species *C. distans* revealed it has an undifferentiated venom duct and likely uses a single venom for defence and prey capture, as previously hypothesised for ancestral species [7]. Transcriptomic analysis identified novel α- and ω-conotoxin-like sequences similar to conotoxins associated with predation in piscivores, suggesting that these sequences might represent ancestral defensive venom in this worm-hunting species. As the venom gland began to compartmentalise, as evidenced by the proto-compartmentalised gland seen in *C. planorbis*, the expression of these sequences was likely localised to discrete duct sections, facilitating the separate evolution of predatory and defensive venoms. Our study supports a pivotal role of venom gland compartmentalisation in guiding the evolution of venom peptides that allowed mollusc- and fish-hunting to evolve in cone snails.

## Figures and Tables

**Table 1:** Conotoxins in *C. distans*.

Name (Superfamily) and Predicted Mass (Da)	Predicted Mature Peptide	Framework	Related Conotoxins	Likely Activity
Dis2 (A)—3582.4	RIAEPNTEEEWNECCKDPSCRNNHMDRCAE	CC-C-C	Di1.1, Di1.2 (*C. distans*)	nAChR antagonists
Dis4 (B1)—1888.9	TITAQEAETARERLSTL	Linear	Conantokin-E1 (*C. eburneus*)	NMDA receptor antagonist
Dis9 (B1)—1234.7	LVGEVEIIVHK	Linear	Conantokin-V (*C. vitulinus*)	NMDA receptor antagonist
Dis14 (B1)—1947.9	QTEEEVEESQEKLEEL	Linear	Conantokin-G (*C. geographus*)	NMDA receptor antagonist
Dis17 (Con-ikot-ikot)—12726.0	GDPTAECCLTLLGCYDTCSHDNTSPDCWGHCKSESTTGCSLDFSLHYCSEFQDCYGPCVTDKDESRCFKACRKEAMIDCLDHGSVECCPGFVNCYQNCRRSREDCITHCLKENC	N/A	Con-ikot-ikot (*C. striatus*)	AMPA receptor antagonist
Dis20 (SFMi2)—2928.9	DCGRDCVGCDNPANCCCGGQTCVNGNKCE	XXVII	Mi045, Mi046 (*C. miles*)	N/A
Dis22 (M---L-LLTVA)—6305.5	DQTASGFLRDDESVFPCNSDRCACLPKEGSTTSYQCQSLEASTDDCVNNECITEDEW	IX	Cl9.5 (*C. californicus),* Turripeptide IX-01	N/A
Dis30 (M---L-LLTVA) (DiXIXA)—4953.8	GCDPTDGCQTTVCETDTGPCCCKPNFTCQISNSGTKSCSCSGQPSDCPV	XIX	N/A	Hyperexcitability/lethargy (dose-dependent)
Dis38 (H)—3984.5	TDVDCGGVSCTFGCCETVNGEKKCKELDCDVTSDTENS	VI/VII	Teretoxin Tsu6.5	N/A
Dis43 (I1)—3378.3	CSYSSCKTESCCTGYLCNSVKSCVDPNSGGRF	VI/VII	Im11.14 (*C. imperialis*)	N/A
Dis45 (L)—2026.8	RCPIACKTCPDENTCIPAP	XIV	Cl14.2 (*C. californicus*)	N/A
Dis46 (M)—3711.5	TCDPYYCNDGKVCCPEYPTCGDSTGKLICVRVTD	VI/VII	Im6.7 (*C. imperialis*)	N/A
Dis49 (M)—1801.8	DVKCIGSCDSTVWHRV	N/A	M-conotoxin 6 (*C. marmoreus*)	N/A
Dis53 (NSDis1)—5430.2	SCEGNDSYCRKPDWISDKPCCDPLVCVCTGPMSGGRRTTCETGKCGPRPSS	XV	N/A	N/A
Dis58 (NSDis3)—3668.3	NACELDSSTGDDCTGTQICCTPSGSMSGECREADEC	VI/VII	Lp7.1 (*C. leopardus*)	N/A
Dis60 (NSG3)—6759.6	DESQCPECRCHDLKNAICDISEACSDEASCPTSPECNNGNCLCKNFHGGRCVSHSQCNDRHC	XXVIII	G125, G126 (*C. geographus*)	N/A
Dis62 (O1)—3509.3	CDPPGYPCELRENDCCDACKIVSQNPNVCSDE	VI/VII	MiK42 (*C. miles*)	ω-conotoxin-like
Dis63 (O1)—3244.3	CLSIGYACGVAISEKCCHVCDNPTGAGTCVYN	VI/VII	Im6.1 (*C. imperialis*)	ω-conotoxin-like
Dis68 (O1)—3205.2	SCAESGLSCDTRPCCDDKTCVRNGRQSMCS	VI/VII	Mr6.11 (*C. marmoreus*)	N/A
Dis70 (O1)—3078.1	CTESGLTCWPTNHDCCSGTCNGTMTTGTCT	VI/VII	Mi024 (*C. miles*)	ω-conotoxin-like
Dis71 (O1)—3087.0	CEDEGSPCQFDSECCSGACTPEGVFDFCE	VI/VII	ω-like peptide (*C. capitaneus*)	ω-conotoxin-like
Dis72 (O1)—2969.1	SCANYHESCASDPCCEGLECIGAQGGGVCI	VI/VII	ArMKLT2-0322 (*C. arenatus*)	N/A
Dis76 (O1)—2789.9	CKGTDAPCDDHDECCEHVCDGVCVED	VI/VII	MgJr94P (*C. magus*)	ω-conotoxin-like
Dis80 (O1)—2503.8	CEDPGEPCGSDHSCCSGSCNHNVCA	VI/VII	ArMKLT2-0322 (*C. arenatus*)	ω-conotoxin-like
Dis81 (O2)—8000.6	DEDCVTEEGDHVEEGKASLLTTATRVPAMMENCTVRLCTVNNGVTEVLTNHENSVDDSLITWNLWTPCHCFI	XIV	N/A	N/A
Dis85 (O2)—4165.4	QCAPDDFQCDVDEDCCNDLECKCFTSTDCTSGYKCRN	VI/VII	Bt15a (*C. betulinus*)	N/A
Dis91 (O2)— 3759.3	CKGASALCEEDGECCSGDCKCMHASGCTNDINLRCAA	VI/VII	Di6.5 (*C. distans*)	N/A
Dis97 (O2)— 3768.4	TCKGRLQSCDHDSECCSPYTCYCGMKQGCNLKCI	XV	Vt15a (*C. vitulinus*)	N/A
Dis98 (O2)—3264.1	CDTWRDPCTYEHECCWQYHCGFRTCE	VI/VII	Di6.10 (*C. distans*)	N/A
Dis102 (O2—contryphan)—1316.5	EFDCPWHPWC	N/A	Contryphan-R-like (*C. virgo*)	Contryphan-like activity
Dis104 (O2)—4409.7	HGGCLNEEGDLVAEDGETIEVECNRCRCEDGDLACTKMACE	IX	Di6.5 (*C. distans*)	N/A
Dis116 (O3)—3535.5	NVDQECIDACQLEDKNCCGRTDGEPRCAKICL	VI/VII	Di6.6 (*C. distans*)	N/A
Dis119 (O3)—3871.5	LVEGACTSPSNCPTGQECCPNKLDEPEGSCANDCPFY	VI/VII	Di6.12 (*C. distans*)	N/A
Dis120 (P)—2780.9	STCPTSCATHMNCWPECTYCTTSGCT	IX	Fla9.1 (*C. flavidus*), TxIXA (*C. textile*), GmIXA (*C. gloriamaris*)	spasmodic
Dis121 (S)—3803.1	SSCSGTCYGSANCDGTCYCREDNCWCTGDSSCACQCA	VIII	Di8.1 (*C. distans),* GVIIIA, RVIIIA	5-HT3 receptor and nAChR antagonists
Dis124 (T)—8350.9	TPSEQNLPGELTPADLEGAETTPEESWYSKIKSGVKHASCKLVGYACDDSETEESLLSKIKGGVEHAACKYVNIACED	XIV	Tr5.4 (*C. terebra*)	N/A
Dis134 (T)—6399.6	APSEPNLQRGLKLGGLKAEPNLQRGLKLGGVKDKLLKVGGNILKGAVQGAVDSLTKEDRKQ	N/A	Vr5.4 (*C. varius*)	N/A

N/A not ascertained.

**Table 2:** MS/MS identification of transcriptomic sequences.

	Sequence
Dis1	MGMRMMFIVFLLVVLATTVVSLRSDRAFNRKNR**RIAEPNTEEEWNECCKDPSCR**NNHLDRCPE
Dis10	MELYTYLYLLVPLVAFHLIQGTGTRSHGGPLTEGRSADVTALKPEPVLLQKSDARSTD**DNGKDKLTRMKRTLKKGGN**MARRQTEEEVEESNETLAEAGKR
Dis102	MKKLTILVLVAAVLLSTQVMVQGDGDQP**ADRNAVPRD**DNPGGTSGKLMRVLQGREFDCPWHPWCG
Dis103	MNKLTMLILVATVLLSIQVMVRG**DEDCVNEEGDLVAEDGETVKVECNBTrCRCDDGDLACTKMACE**
Dis11	MQLYTYLYLLVPLVAFHLIQGT**GTRGHGGALTEGRSADVT**ALKPEPVLLQKSDARSADDNGKDKLTRMRRTLKNKGNMARRQTEEEVEESQEKLEELGKR
Dis118	MSGLGIMVLTLLLLVPMATSQQDGGEKQAMQRDAINAAPGTSITRRETD**QECIDTCEQE**DKKCCGRTNGEPVCAKICFG
Dis121	MMSKMGAMFVLLLLCPLAS**NQQEGDIKA**RRTFWKRDLYGDLAGRSSCSGTCYGSANCDGTCYCREDNCWCTGDSSCACQCA
Dis125	MLCLPVFIILLLLASPAVT**TPSEQNLPGELTPADLEGAETTPEESWYSKIKGGVKHASCKLVGYACDDSETEESLLSKIKGGVEHAACKYVNIGCED**
Dis126	MLCLPVFIILLLLASPAVTTLSEQNLPGELTPADLE**GAETTPEESWYSKIK**SGVKHASCKLVGYACDDSETEESLLSK**IKGGVEHAACKYVNIACED**
Dis127	MLCLPVFIILLLLASPAVTTPSEQNLPGELTPADLE**GAETTPEESWYSKIK**SGVKHASCKLVGYACDDSETEESLLSKIKG**GVEYAACKYVNIACED**
Dis128	MLCLPVFIILLLLASPAVTTPSEQNLPGELTPADLE**GAETTOEESWYSKIKGGVK**HASCKLVGYACDDSETEESLLSKIKGSVEH**AACKYVNIGCED**
Dis132	MLCLPVFIILLLLASPAVTTPSEQNLPGE**LTPADLEGAETTPEESWYSKIKGGVK**HASCK**LVGYACDDSETEESLLSK**IKGVSNMLRANTLI
Dis133	MLCLPVFIILLLLAAPAVTAPSEPNLQRGLKLGGLKAEPNLQRGLKLGGVKEGLLKVGASAIKGAVNGALN**SITKEDRKK**
Dis135	MLCLPVFIILLLLAAPAVTAPSEPNLQRGLKLGGLKAEPNLQRGLKLGGVKDKLLKVGGNIFKGAVQGAVD**SLTKEDRKQ**
Dis16	MLRLIITAVLASACLALPHRR**DAAPADMGALKPFEQQMQPM**GMPGSMAGMQGMPGQQ**AMPGGMLGNQLMPFGPGMGMGAGY**RRAADHNQEKRDLPLT
Dis18	MNMWMTPSVLVVVVFTATVVCSTEDERLTRQRRGDPTAECCLTLLGCYDTCSHDNTSPDCWGHCKSESTTGCSLDFSLHYCSEFQDCYGPCVTDKDESRCFKACRKE**AMIDCLDHGSVE**CCPGFVNCYQNCRRSREDCITYCLKENC
Dis2	MGMRMMFIVFLLVVLATTVVSLRSDRAFNRKNR**RIAEPNTEEEWNECCKDPSCRNNHMDRCAE**
Dis20	MNFYLLLTVTLLLASFTGGDARRIQGMDIYRHFVRRDCGR**DCVGCDNOANCCCGGQTCVNGNK**CE
Dis21	MNFYLLLTVTLLLASFTGGDARRIQGMDIYRHFVRR**DCGKDCVGCDNPANCCCGGQTCVNGNKCE**
Dis26	MGFRQLVTVGLLLTFFMSTDASHADQTESGFLR**DDETVFPCNSDRCACLPK**EGSTTSYQCQSLEASTDDCVNNECITEDEWSGRR
Dis27	MGFRQLVTVGLLLTFFMSTDASHADQTESGFLR**DDETVFPCNSDRCACLPK**EGSTTSYQCQSLETSTDGCVNNECVTEDEW
Dis28	MRFLLRLTVALFLTWFTETDAAAIGKR**EVHQVILGEPLTNYATVPPDAFQQKLPEIILGQPLME**YQSTESPEVLS
Dis29	MRFLLRLTVALFLTWFTETDAAAIGKR**EVHQVILGEPLTN**YVPPDAFQQK**LPEIILGQPLME**YQSTESPEVLS
Dis3	MQLYTYLYLLVPLVAFHLIQ**GTGTLGHGGALT**EGRSADATAPKPEPVLLQKSDARSADNSKDKLTQMKRTLKKQGHIARTITAEEAERNRERMSTLGKR
Dis30	MSTLGILLLIALLLPLANPAETGDGQAMPRTRNLRSLSFGRTLRRLEKR**GCDOTDGCQTTVCETDTGOCCCKONFTCQISNSGTKSCSCSGQOSDCOV**
Dis43	MKLSVALLLIVLLLPVVAGEKESGDHVLKKR**CSYSSCKTESCCTGYLCNSVKSCVDPNSGGRF**GK
Dis44	MKLSVALLLIVLLLPVVAGEKESGDHVLKKR**CSYSSCKTESCCTGYLCNSVKDCVDPNSGGRF**GK
Dis52	MMTKLGAVTLLSLVIIPQVLLQQHQDG**IVDVKSMQRNK**GRTAAGSVLSHSLRSTNNEYDAKHER**SCEGNNSYCRKPDWVGDKPCCSPLVCVCTGTMSG**GRRTTCKRAK**CGOHOSS**K
Dis54	MMTKLGAVTLLSLVIIPQVLLQQHQDSIADVKSMERNKGRTAAGSVLSHSLRSTNNEYDAKHKR**SCEGNDSYCRKPDWISDKOCCDPLVCVCTGOMSGGRRTTCETGK**CGPRPSSK
Dis55	MMTKLGAVTLLSLVIIPQVLLQQHQDSIADVKAMERNKGRTAAGSVLSHSLRSTNNEYDTKHKR**SCEGNDSYCRKPDWISDKPCCDPLVCVCTGOMSG**GRR**TTCETGKCGOROSS**K
Dis56	**MMTKLGAVTLLSLVIIPQVLLQQHQDSIADVK**AMERNKGRTAAGSVLSHSLRSTNNEYDAKHKRSCEGNDSYCRKPDWISDKPCCDPLICVCTGPMSGGRRTTCETGKCGPRPSSK
Dis57	MIQALASMAWTSMLCSADQVSTSPSVPTFVMV**LMATVLLTGIMETEART**LFQMIARR**SSDYPCAGTFADCRGQPDGATCCDTGYCQGNVCHY**
Dis59	MQLSVILFVLLLT**MPLFNGSVLNAINGRKT**FERNDRSTDSSQMFEKRCPTACK**SCSOOGTCQPV**R
Dis60	MKMYLCLAVVLLLASTIVDSALLDKTETLRNWRRKGRDESQCPECR**CHDLK**NAICDISEACSDEASCPTSPECNNGNCLCK**NFHGGR**CVSHSQCNDRHC
Dis61	MKMYLCLAVVLLLASTIVDSALLDKTETLRNWRRKGRDESQCPECRCHELKNAICDISEACNDEASCPTSPGCNNGNCLCK**NFHGGRCVSHSECNDR**HC
Dis64	MKLTYALIVAVLFLTACQVITTDDSRDKQDLLAMLFSKKRNSRDSKWLTKRCLSIGY**ACGVAISEKCCHVCDNOTGAGTCVYN**
Dis65	MKLTYALIVAVLFLTACQVITTDDSRDKQDLLAMLFNKKRNSRDSKWLTKRCLSIGYACGVAISE**KCCHVCDNPTGAGTCVYN**
Dis66	MKLTYALIVAVLFLTACQLITTDDSRDKQDLLAMLFNKKRNSRDSKWLAKRCLSIGYACGVAISE**KCCHVCDNPTGAGTCVYN**
Dis67	MKLTYALIVVVLFLTACQLLTADYSRDKQEYPTMRFRDQMRNAKGPKWIR**SCAESGK**SCDTK**VCCDDMγCIGTPGGSMCN**G
Dis72	MKLTCVLVVAVLFLTACQFNTADDSRNKQEYRAARLRVGMQKSNGFRSCANYHESCASDPCCE**GLγCIGAQGGGVCI**
Dis73	MKLTCVLVVAVLFLTACQFNTADDSRNKQEYRAARLRVGMQKSKGFRSCANYHESCASDPCCE**GLECIGAQGGGVCI**
Dis87	MKELMILILVATALLSIQVMVRGDGEKPLMGGIKRNAAAGLSALIRGKRCKGTSAICEEDGECCSDDCK**CMIASGCSNHINR**RCAA
Dis88	MKELMILILVATALLSIQVMVRGDGEKPLMGGIKRNAAAGLSALIRGKRCKGESAICEEDGECCSDDCK**CMIASGCSNHINR**RCAA
Dis89	MKELMILILVATALLSIQVMVRGDGEKPLMGGIKRNAAAGLSALIRGKRCKGASAICEEDGECCSDDCK**CMIASGCSNHINR**RCAA
Dis90	MKELMILILVATALLSIQVMVRGDGEKPLMGGVKRNAAAGLSALIRGKRCKGTSAICEEDGECCSDDCK**CMIASGCSNHINR**RCAA
Dis93	MKELMILILVATTLLSIRVMVRGDGEKPLMGGIKRNAAAGLSALIRGKRCKGASALCEEDGECCSGDCK**CMHASGCTNDINLR**CAA
Dis95	MKELMILILVATALLSIQVMVRGDGEKPLMGRIKRNAAAGLSALIRGKRCKGTSALCEEDDECCSGDCK**CMIASGCTNDINLR**CAA
Dis96	MKELMILILVATALLSIQVMVRGDGEKPLMGRIKRNAAAGLSALIRGKRCKGASALCEEDGECCSGDCK**CMIASGCTNDINLR**CAA

MS validated sequences (>99% confidence) are bolded. Identified PTMs are O = Hydroxyproline, Btr = Bromotryptophan, γ = γ-Carboxyglutamic acid. Underlined residues indicate oxidation.

## Data Availability

All available data are published in the manuscript and Supplementary Data.

## Supplementary Materials

The following supporting information can be downloaded at: www.mdpi.com/article/10.3390/toxins14030226/s1, Figure S1: LC-ESI-MS analysis of the four duct sections from *C. distans* specimen 2.; Figure S2: O1 and O2 superfamilies in *C. distans*; Figure S3: O1 and O2 superfamilies in *C. distans*; Figure S4: Conopeptides (A) and divergent superfamilies (B) in *C. distans*; Figure S5: Novel superfamilies in *C. distans*. (A) and (B) indicate novel superfamilies reported only in *C. distans* and novel superfamilies also reported in other species.
