# Supplementary material for: Venomics Reveals a Non-Compartmentalised Venom Gland in the Early Diverged Vermivorous Conus distans
Source: Toxins (Basel). 2022 Mar 19;14(3):226. doi: 10.3390/toxins14030226 (PMC8949452; doi:10.3390/toxins14030226)
Supplement: Supplementary file 1 [file toxins-14-00226-s001.zip › toxins-1621661-supplementary.pdf]

Supplementary Materials: Venomics Reveals a Non-Compartmentalised Venom Gland in the Early Diverged Vermivorous *Conus distans*

Jutty RaJan Prashanth, Sebastien Dutertre, Subash Kumar Rai and Richard J. Lewis

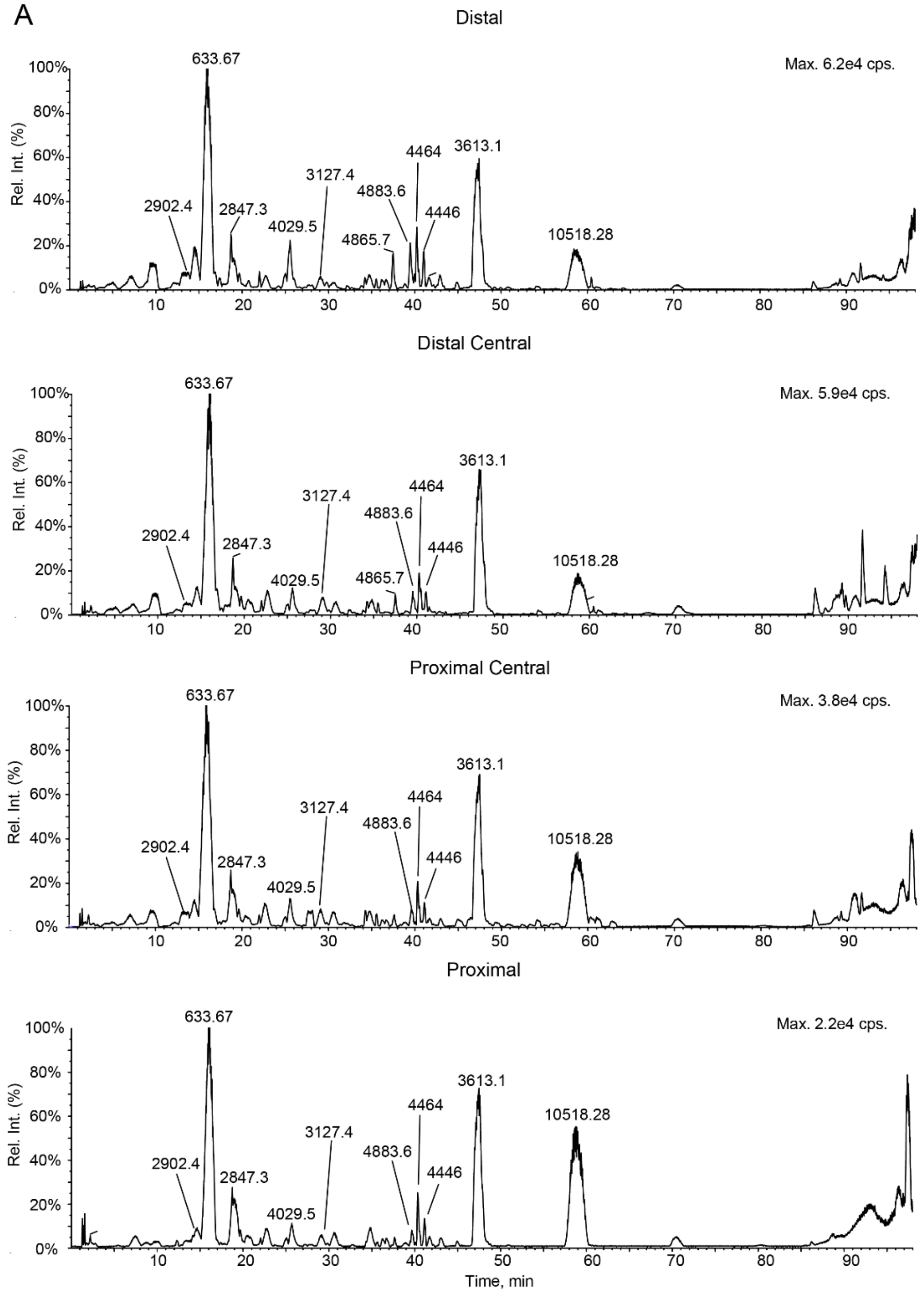

Figure S1. LC-ESI-MS analysis of the four duct sections from *C. distans* specimen 2.

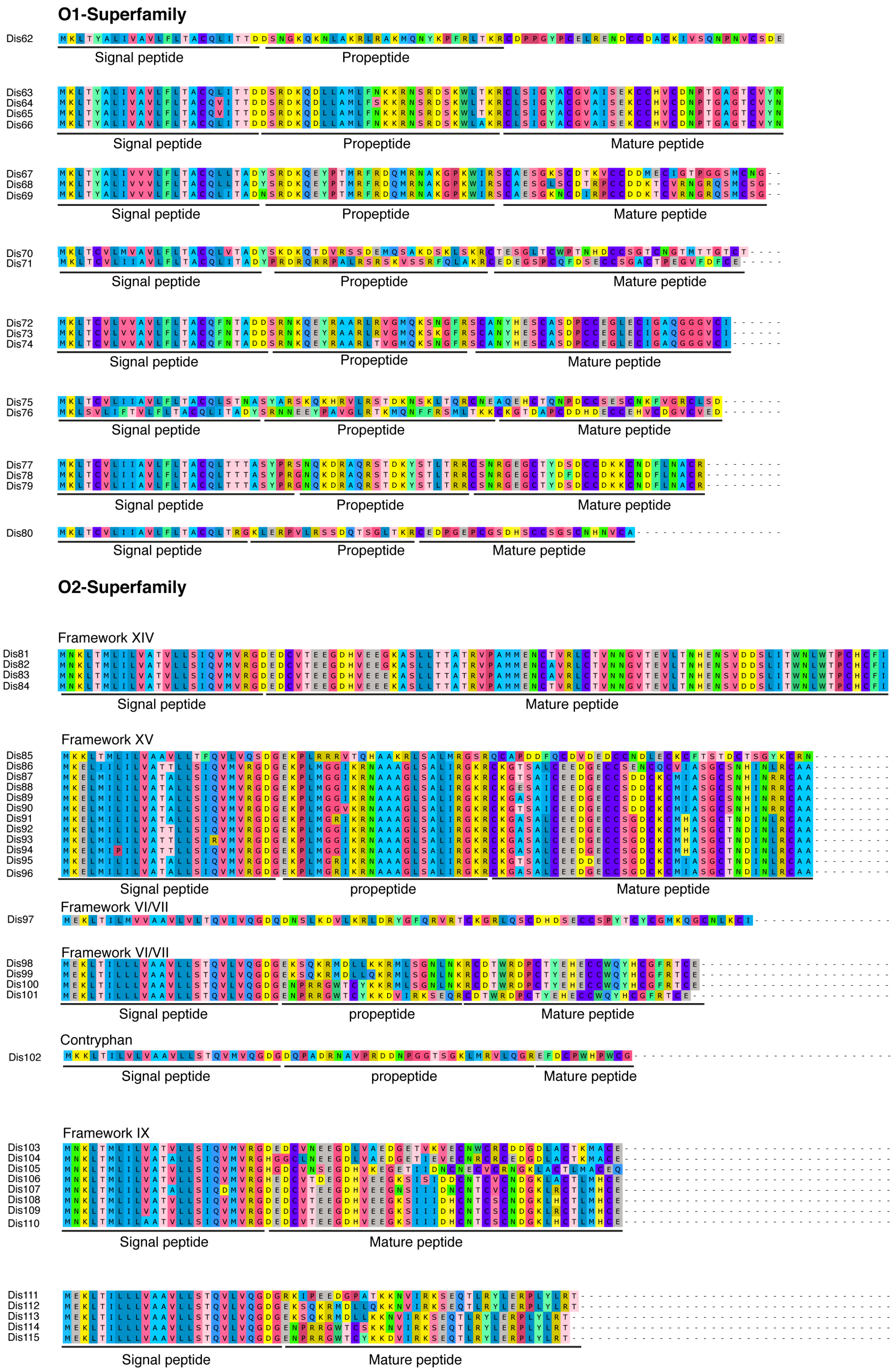

Figure S2. O1 and O2 superfamilies in *C. distans*.

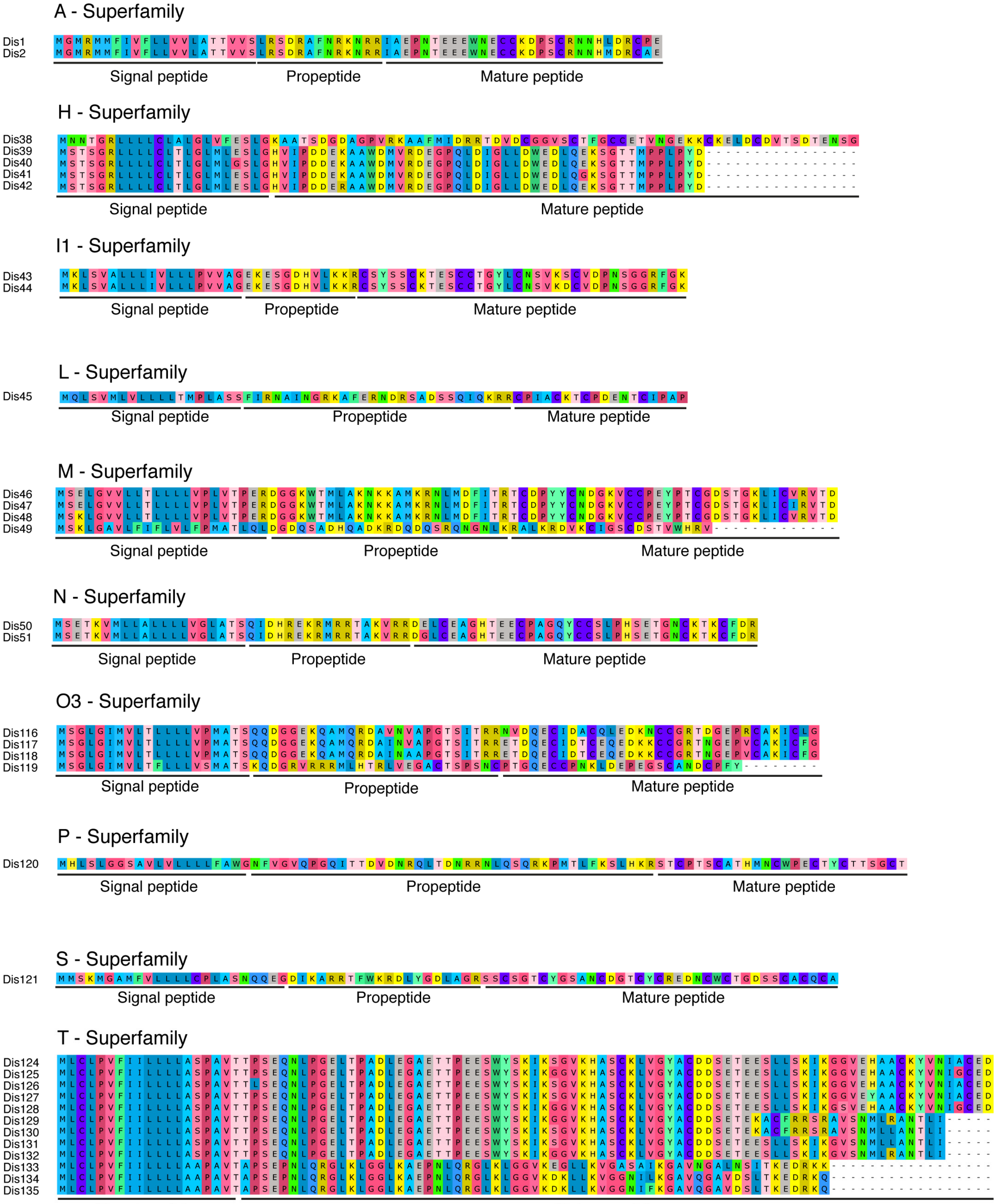

Figure S3. Other known superfamilies in *C. distans*.

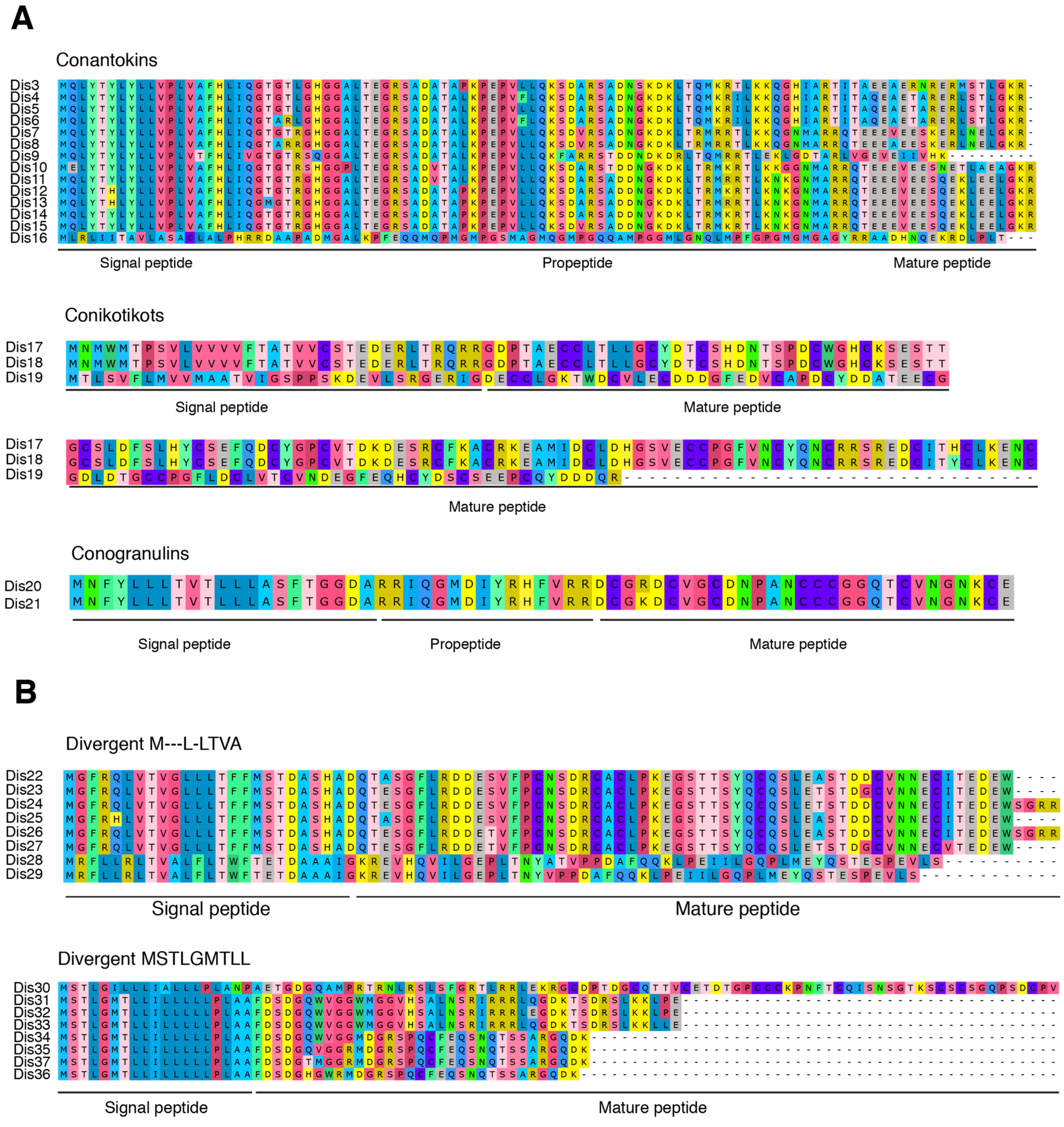

Figure S4. Conopeptides (A) and divergent superfamilies (B) in *C. distans*.

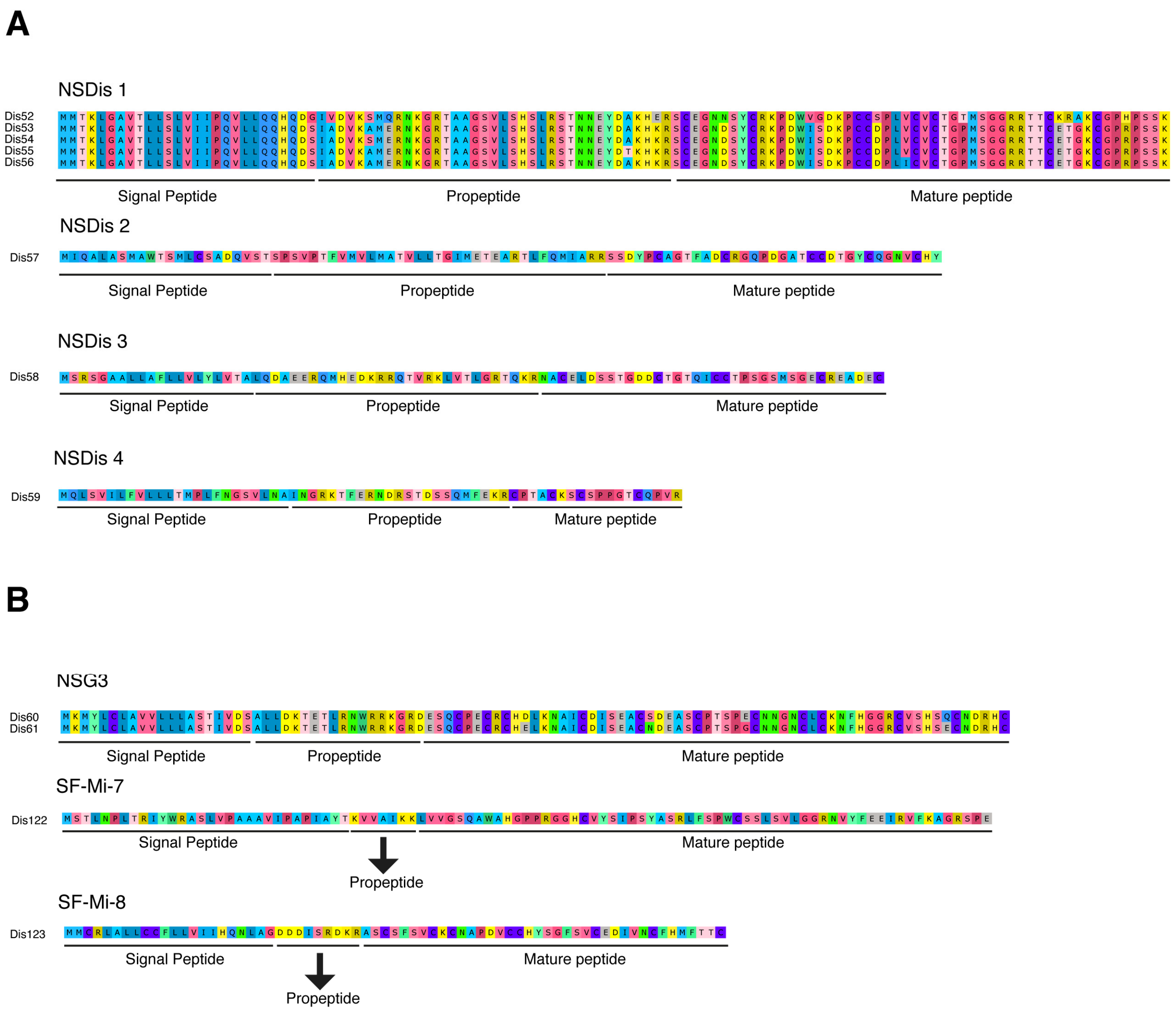

Figure S5. Novel superfamilies in *C. distans*. (A) and (B) indicate novel superfamilies reported only in *C. distans* and novel superfamilies also reported in other species.
